# Modulations of the P3b effect as a function of bilingual language experience

**DOI:** 10.1038/s41598-026-47785-z

**Published:** 2026-05-25

**Authors:** Nancy E. Rodas De León, Heather Bortfeld, Kristina C. Backer

**Affiliations:** 1 Department of Psychological Sciences, University of California, Merced, 5200 North Lake Road, Merced, CA 95343 USA; 2 Department of Cognitive and Information Sciences, University of California, Merced, 5200 North Lake Road, Merced, CA 95343 USA; 3 Health Sciences Research Institute, University of California, Merced, Merced, CA 95343 USA

**Keywords:** Degree of bilingual engagement, P3b, Attention allocation, Electrophysiology, Young adults, Neuroscience, Psychology, Psychology

## Abstract

Recent theoretical accounts propose that variability in bilingual language experience can drive neuroplastic modifications in attentional systems. In the present study, we empirically evaluated this proposition by examining whether continuous variation in bilingual language experience predicts neural activity associated with the allocation of attention to salient environmental stimuli. To this end, we recorded electroencephalography (EEG) and response time data from young adults with diverse language backgrounds as they completed an active visual oddball paradigm known to elicit the P3b component. Language experience was quantified using composite factor scores derived from the Language and Social Background Questionnaire, which served as an index of participants’ cumulative bilingual language engagement. Because childhood family socioeconomic status (SES) is known to influence neural development and adult cognitive functioning, we also controlled for childhood family SES. Although neither bilingualism nor childhood family SES was associated with behavioral performance, greater bilingual language experience was positively associated with the magnitude of the P3b effect. These findings indicate that, even after accounting for childhood family SES, lifelong bilingual language engagement may shape the neural mechanisms supporting attention allocation during dynamic stimulus tracking.

## Introduction

On a global scale, bi-/multilingualism is the norm rather than the exception. Even within the United States, approximately 20% of the population speaks a language other than English at home^1^. Because bilingual language experience is dynamic, changing across contexts and stages of life^2–6^, it is critical to understand how cumulative language experience shapes the neural mechanisms that underlie core cognitive processes. Indeed, prior research has reported that bilingualism affects a range of cognitive mechanisms, including attention, working memory, conflict monitoring, and inhibitory control; however, consistent replication of these findings has proven challenging^7–17^. Researchers have attributed these challenges, at least in part, to how bilingualism is conceptualized^6,18–22^, issues of task impurity^23–25^, unaccounted confounding variables such as socioeconomic status^26,27^, as well as the lack of clear, theoretically motivated predictions regarding the cognitive consequences of bilingual engagement^28^.

Notably, in response to these theoretical difficulties, Pliatsikas proposed the Dynamic Restructuring Model (DRM) to better explain the observed structural adaptations in the brain as a consequence of bilingual language experience^29^. The DRM is a three-stage model that proposes a time-course for structural adaptations, suggesting that these modifications are dynamic and depend on the quantity and quality of second language (L2) exposure and usage^29^. The three stages are: initial exposure, consolidation, and peak efficiency. In Stage 1, there is an increase in cortical gray matter to meet the initial demands of language learning, such as lexical acquisition. As a bilingual gains more experience and immersion, during Stage 2 the brain shifts from learning to efficiently handling language control; this shift is mediated by increases in the grey matter of subcortical structures and in long-range white matter connectivity, while the cortical regions that expanded in Stage 1 undergo “renormalization” (e.g., via synaptic pruning). In the final stage, individuals with the most bilingual experience achieve automation in language processing, as indexed by adaptations that move further toward posterior and subcortical networks, and the brain requires fewer cognitive resources to meet processing demands. In sum, the DRM proposes three stages of structural plasticity that the brain undergoes, as bilingual language experience shifts from initial learning toward automatic control and efficiency^29^.

As the DRM focuses specifically on how the brain changes over time, other models focus on how different factors interact to speed up or slow down these neuroplastic effects^30^. Specifically, DeLuca and colleagues introduced the Unifying the Bilingual Experience Trajectories (UBET) model to more thoroughly map observed associations between neurocognitive modifications and aspects of bilingual experience, such as intensity and diversity of language use, language switching, relative proficiency, and duration of bilingual experience^30^. Interestingly, the UBET model predicts that as bilingual language engagement increases, the brain becomes more efficient, and consequently, in terms of EEG/ERP data, modifications in response to bilingual language engagement may manifest as earlier and larger N2 and P3 responses^30,43,44^. These findings would indicate that with increasing bilingual language engagement, the brain detects conflict faster (as indexed by the N2) and more efficiently allocates attention to important stimuli and automatically categorizes stimuli (as indexed by the P3b component)^30^. DeLuca et al. argue that these adaptations are not dependent on the duration of language use alone, but rather that increased intensity and diversity of the use of two languages may accelerate the emergence of neurocognitive adaptations. Thus, this framework enables theoretically-driven predictions describing how variation in bilingual language engagement shapes neurocognitive processes.

While the DRM and UBET frameworks emphasize the neurocognitive modifications that occur in response to bilingual language engagement, other recent frameworks have focused on characterizing *which* cognitive mechanisms are modified. For instance, Bialystok recently proposed the adaptation view, which posits that bilingual language experience continually tunes attentional mechanisms to meet the dynamic demands of managing two language systems^31,32^. Within this framework, neurocognitive adaptations vary continuously with the intensity of bilingual experience: the greater one’s language proficiency, use, and engagement across contexts, the stronger the corresponding cognitive and neural modifications^31,32^. This perspective suggests that bilingual language experience can drive experience-dependent neuroplasticity, through which an individual’s attentional processing is shaped by patterns of language use across the lifespan^31,32^. Together, these theoretical frameworks describing experience-dependent adaptations in bilinguals motivate the present study’s focus on how variation in lifelong bilingual engagement relates to attentional processes.

To investigate this question, we examined the P3b event-related potential (ERP) component, which is widely interpreted as reflecting the allocation of attention to task-relevant stimuli^33,34^. The P3b is a positive-going ERP component observed approximately 300–600 ms post-stimulus onset in healthy adults^35–37^, and it is reliably elicited in young adults using an active visual oddball paradigm. In this task, participants view a sequence of visual stimuli comprising rare (oddball) target stimuli presented among frequent (standard) non-target stimuli^35–37^. Before each block, participants are shown the target stimulus and are instructed to press a button for targets (oddballs) and another for non-targets (standards), maintaining the target representation in working memory.

Because oddball stimuli occur less frequently than standard stimuli, it has been proposed that the P3b reflects enhanced attentional allocation to these rare events^33,34,38^. Consistent with this view, the P3b exhibits a larger (i.e., more positive) amplitude for oddball stimuli than for standard stimuli^35–37,39^. Therefore, the P3b component elicited in the present study provides an appropriate neural correlate for examining how bilingual language engagement influences the allocation of attention to task-relevant environmental stimuli. While prior research has examined the relationship between bilingualism and attention^40–43^, the neural dynamics underlying this association remain a topic of debate. Thus, the present study adopts a novel approach by relating variation in bilingual language engagement to the P3b component elicited during the active visual oddball task. In doing so, it aims to elucidate how experience-dependent adaptations in bilingual individuals influence attentional processes.

The P3b component can be elicited by a range of experimental tasks, and the interpretation of these modulations (and the specific cognitive processes they are thought to reflect) varies across paradigms. Within bilingual cognition research, for example, P3b modulations have been primarily examined in tasks assessing inhibitory control and conflict monitoring, such as Stroop, Simon, and Flanker paradigms^11,17,44,45^. Consistent with earlier behavioral studies, initial ERP investigations of bilingual effects relied on categorical comparisons between monolinguals and bilinguals, often reporting group differences in P3b amplitude^46–50^. Although the group-level findings suggest that bilingualism can influence P3b-related conflict monitoring and inhibitory mechanisms, binary comparisons miss the substantial variability inherent in individuals’ unique bilingual language experiences.

Increasingly, the field recognizes bilingualism as a multidimensional construct encompassing language use, attitudes, and proficiency, placing individuals along a continuum rather than within discrete categories^3,6,19–22,51–53^. Adopting this perspective enables researchers to examine how lifelong bilingual experience shapes cognitive processing in a context-sensitive manner^27,54,55^. Continuous measures of bilingual experience offer greater sensitivity; however, characterizing language experience in this way is not without limitations, including measurement noise and reliance on self-reports, which may not always be accurate^56^. Moreover, measuring bilingualism as a continuous construct is inappropriate for some research questions and study designs, such as studies with smaller samples or special populations (e.g., infants or children with atypical development) or when the aspect of bilingualism of interest is inherently binary (e.g., enrollment in a dual language immersion program), in which case categorical approaches may be more appropriate. As such, both continuous and categorical approaches have their respective strengths and limitations, and the choice between operationalizing bilingualism as a dichotomous or continuous variable should be guided by the specific research questions and populations under investigation. Critically, our approach of characterizing bilingualism along a continuum reframes the central question such that, rather than asking whether bilingualism affects cognition, it focuses on determining how, when, and for whom bilingual language engagement shapes cognitive functioning.

Despite this, relatively few electrophysiological studies examine the effects of bilingual language engagement while also contextualizing individuals’ early life socioeconomic status. However, when investigating the effects of bilingualism on cognitive processing, whether measured behaviorally or neurally, it is important to take into account variation in individuals’ socioeconomic status (SES). For instance, prior studies suggest that when examining differences between language groups (i.e., bilinguals vs. monolinguals), controlling for SES may attenuate the behavioral advantages typically observed in bilingual groups^26,57^. However, other work suggests that while bilingualism and higher SES are independently correlated with better behavioral performance on cognitive processing tasks, no interaction between the two factors was observed^58^. This finding suggests that the contributions of bilingualism and SES operate independently of one another.

Importantly for the context of this study, the neural mechanisms indexed by the P3b effect are particularly sensitive to childhood family SES^59,60^. For instance, Isbell and colleagues found that higher childhood parent education, a common proxy for family SES, was associated with larger ΔP3b amplitudes (i.e., the difference in P3b amplitude between rare – frequent stimuli on an oddball task) in young adulthood, despite no observable differences in behavioral performance (i.e., *d*′). The authors suggested that early-life SES can have lasting effects on neural functioning, as indexed by the P3b, providing compelling evidence for a link between early socioeconomic context and adult cognitive control^61^. Together, prior work underscores the importance of considering childhood parent education when investigating how bilingual engagement shapes adult neural processing.

Building on the continuum-based perspective and accounting for SES, recent work has examined how the extent of bilingual experience and SES relate to neural activity. However, these studies vary considerably in their participant populations, task designs, and approaches to measuring SES. For example, Jiao et al. investigated whether P3b amplitudes elicited by a spatial Stroop task varied as a function of bilingualism in Mandarin Chinese-English bilinguals who had learned English primarily through classroom instruction^62^. In their study, bilingualism was quantified via L2 (English) proficiency and frequency of language switching, while SES was indexed using a composite of parental education, occupation, and family income. Their findings indicated that higher L2 proficiency, measured continuously, predicted larger P3b responses, whereas language-switching frequency and SES did not; none of the measures were associated with behavioral response times^62^. In contrast, Pereira Soares and colleagues examined bilingualism-related modulations of the P3b elicited by an N-back task in a sample of L2 learners (i.e., German-English) and early Italian-German-English trilinguals. All participants were German-dominant and had acquired English through formal classroom instruction^63^. Maternal education alone was used as the SES indicator. In this study, no associations emerged between P3b amplitude and bilingualism, despite the inclusion of several other indices, including duration of dual-language use, age of first language (L1) and L2 acquisition, and use of the non-societal language in home and social contexts. In this study, effects of maternal education on behavioral or neural outcomes were not reported.

Taken together, these studies, which involved distinct bilingual populations and employed different experimental paradigms to elicit the P3b, produced divergent results. Consequently, it remains unclear whether (and under what conditions) bilingual language experience modulates attentional allocation to salient and task-relevant events, as indexed by the P3b elicited by the active visual oddball task used here. By employing a task that targets attentional engagement rather than conflict monitoring or working memory updating, the present study provides the opportunity to test whether the influence of variation in bilingual experience generalizes across other cognitive processing domains. These differences in task design and sample characteristics provide critical leverage for assessing the generality of bilingualism-related neural adaptations.

In the present study, we adopted a multimodal approach to investigate how variation in bilingual language experience relates to both behavioral and neural markers of attentional processing among young adults from diverse linguistic backgrounds in the Western United States. To accurately quantify bilingual language experience, we used the Language and Social Background Questionnaire (LSBQ) ^64^. Furthermore, because childhood parent education has been linked to neural processes indexed by the P3b effect in a visual oddball in adulthood^61^ and SES has been identified as a potential confounding factor in prior research^26,27^, we statistically controlled for SES using parental educational attainment during participants’ upbringing. To elicit the P3b, we employed the Active Visual Oddball task^35^ and isolated the P3b effect using a difference wave approach (ΔP3b = Rare Oddball - Frequent Standard). We hypothesized that, if bilingual language use enhances attentional processing as postulated by previous work^31,32^, then incremental increases in bilingual language experience would be associated with faster ΔResponse Times (Rare Oddball Response Time - Frequent Standard Response Time) and larger ΔP3b amplitudes. A significant association between bilingual language experience and ΔP3b amplitude would therefore provide evidence of experience-dependent neural adaptation, whereby lifelong use of two languages shapes attentional processes in young adulthood.

## Results

### Descriptive statistics

Descriptive statistics for participant age, participant education, highest childhood parent education, L2 age of acquisition (AoA), bilingualism composite factor score, *d*′, Response Times (RT), and P3b mean amplitudes are reported in Table 1.

**Table 1:** Descriptive statistics.

Variable	Mean	SD	Min	Max
Age	22.24	2.84	18.83	30.12
Participant education	13.27	1.58	12	18
Childhood parent education	12.11	4.03	4	20
L2 AoA	5.65	5.24	0	15
Composite score	3.85	5.45	-6.48	13.91
*d*′	3.38	0.68	1.89	5.15
Frequent Standard RT (ms)	368.91	103.98	150.90	698.50
Rare Oddball RT (ms)	427.33	119.10	206.80	721.70
ΔRT Effect (ms)	58.42	130.15	-249.30	317.30
Frequent Standard P3b mean amplitude (µV)	5.75	2.74	-1.74	11.06
Rare Oddball P3b mean amplitude (µV)	10.54	4.35	1.06	22.46
ΔP3b mean amplitude (µV)	4.79	2.85	-1.30	13.09

(*n* = 70) Participant education is in years; Childhood parent education: Highest parent education level completed in years. L2 AoA: second language age of acquisition. Composite score: overall degree of bilingualism; Larger (more positive) scores derived from the LSBQ indicate greater levels of overall bilingual language engagement. Behavioral performance: *d*′ where higher scores indicate better overall performance on the task. RT: median response time. ms: milliseconds (computed within each participant). ΔRT Effect: participant-level difference score calculated as median Rare RT − median Frequent RT, then averaged across participants. ΔP3b: difference scores (Rare Oddball – Frequent Standard waveforms) extracted over the centroparietal cluster (Pz, P3, P4, CP1, CP2, CP6) in microvolts (µV).

### Task-elicited effects on neural and behavioral outcomes

First, we conducted paired-samples t-tests to determine whether there were general effects of stimulus condition (Rare Oddball vs. Frequent Standard) on P3b amplitude and RT across all subjects. As expected, P3b amplitudes were significantly larger (more positive) for Rare Oddball trials than for Frequent Standard trials, *t*(69) = 14.06, *p* < .001. In addition, participants were significantly slower to respond to Rare Oddball stimuli than to the Frequent Standard stimuli, *t*(69) = 3.76, *p* = .00036. Accuracy (*d*′) was positively correlated with RT on Frequent Standard trials, such that individuals with higher *d*′ scores responded slower to Frequent Standard stimuli. In contrast, *d*′ was not correlated with RT on Rare Oddball trials, nor was the ΔRT (Rare Oddball RT – Frequent Standard RT) correlated with the corresponding *d*′ difference score. Furthermore, none of these behavioral measures were correlated with Rare Oddball, Frequent Standard, or ΔP3b mean amplitudes (see Table S1 in the Supplementary Materials for zero-order correlations).

### Multiple regression results: bilingual language experience

A summary of the separate multiple regression analyses for behavioral performance and ΔP3b amplitude is presented in Table 2. The first regression analysis indicated that neither childhood parent education nor the degree of bilingual language engagement significantly explained the variance in behavioral performance. This pattern of results held regardless of whether we analyzed RT per condition separately (i.e., Rare Oddball RT and Frequent Standard RT) or *d*′ as the dependent behavioral measure (see Table S2 in the Supplementary Materials for a summary of these additional behavioral analyses).

**Table 2:** Summary of multiple regression analyses for behavioral performance and ΔP3b mean amplitude (µV).

Variable			*B*	*SE B*	*β*	*p*	95% CI	*R* ^2^
Model 1: ΔRT Effect (Rare Oddball RT minus Frequent Standard RT)								
								0.035
Parent education			-6.31	16.96	− 0.049	0.71	[ -40.17, 27.55]	
Composite score			-16.75	17.26	− 0.13	0.34	[-51.21, 17.70]	
Composite score^2^			14.23	16.43	0.11	0.39	[-18.56, 47.03]	
* Model Fit*:			*F*(3, 66) = 0.81, *p* = .49, *R*^2^ = 0.035					
Model 2: ΔP3b Mean Amplitude (µV)								
								0.21
Parent education			0.90	0.34	0.32	0.011*	[0.21, 1.59]	
Composite score			1.38	0.35	0.48	0.00020*	[0.68, 2.08]	
Composite score^2^			0.26	0.34	0.091	0.45	[-0.41, 0.92]	
* Model Fit*:			*F*(3, 66) = 5.91, *p* = .0012, R^2^ = 0.21					

Bonferroni-corrected alpha threshold = 0.025. *p < .025. 95% CI: 95% confidence interval for the unstandardized coefficients. Parent education: the highest parent education completed in years. Composite score: overall degree of bilingual language experience; Larger (more positive) scores derived from the LSBQ indicate greater bilingual language engagement. RT: median response time in milliseconds (computed within each participant). ΔRT Effect: participant-level difference score calculated as median Rare RT − median Frequent RT, then averaged across participants. ΔP3b: difference scores (Rare Oddball – Frequent Standard waveforms) in microvolts (µV), more positive values suggest larger neural responses of P3b.

The second regression analysis indicated that the degree of bilingualism significantly contributed to the variance in ΔP3b amplitude, even after controlling for childhood parent education. Similarly, childhood parent education also significantly accounted for variance in ΔP3b amplitude when controlling for bilingual language experience. Together, parental educational attainment and the degree of bilingualism explained 19% of the variance in ΔP3b amplitude. The variance inflation factor across regression models was ≤ 1.27, which is below 10, indicating an acceptable level of multicollinearity^65^.

**Fig. 1:**
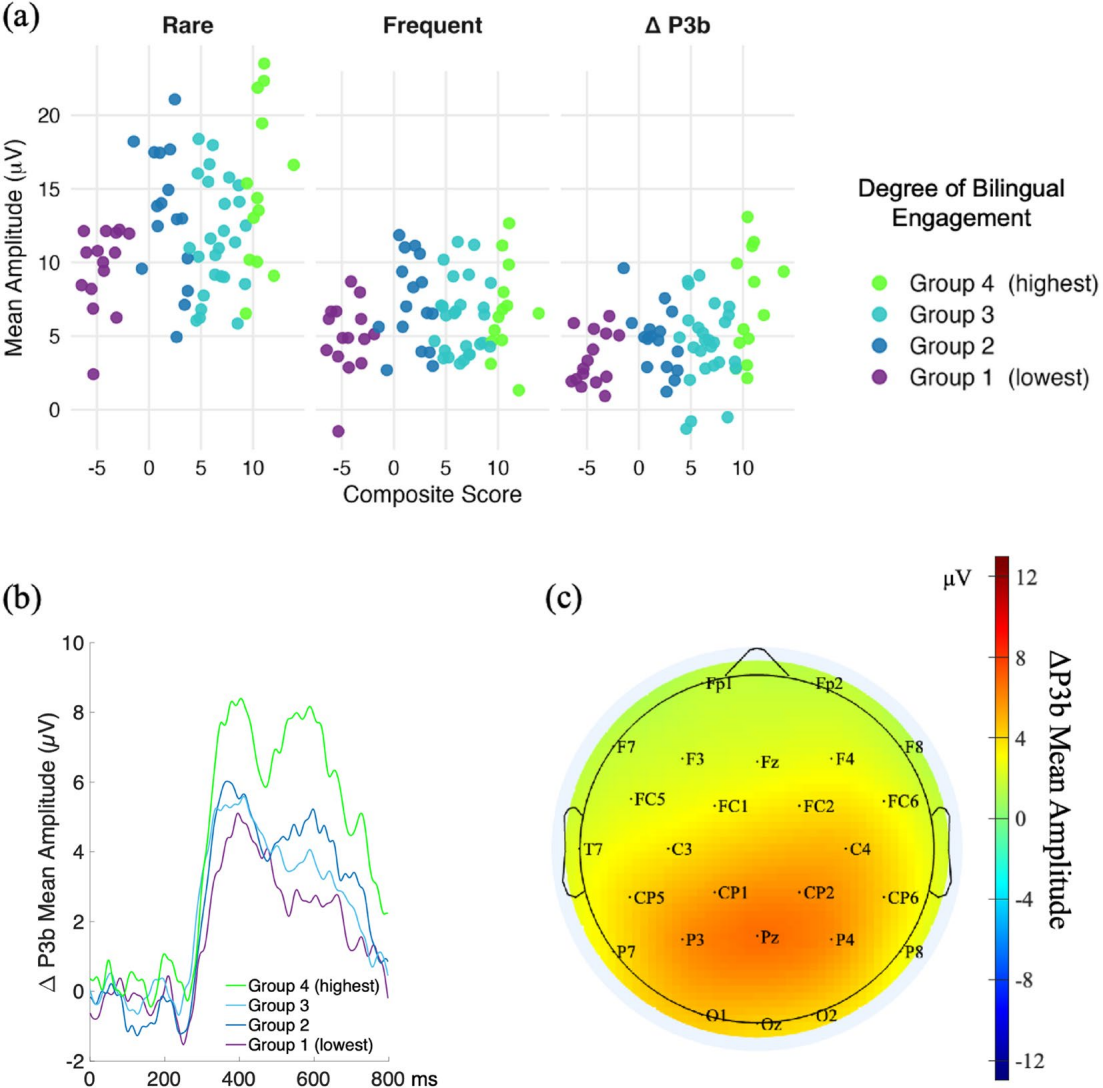
Relationship between bilingual language experience and P3b Mean Amplitudes **(a)** Scatter Plots of Rare Oddball, Frequent Standard, and ΔP3b Mean Amplitudes by Bilingual Language Experience,**(b)** Grand Average Rainbow ERP Plots Over the Centroparietal Cluster by Degree of Composite Scores. ΔP3b was measured between 300–600 ms post-stimulus onset over the centroparietal cluster (Pz, P3, P4, CP1, CP2, and CP6). Group 1 (lowest bilingual experience; *n* = 15) with composite scores from − 7.05 to − 1.61; Group 2 (next lowest; *n* = 16) with scores from − 1.60 to + 3.85; Group 3 (moderately high; *n* = 26) with scores from + 3.86 to + 9.30; and Group 4 (highest; *n* = 13) with scores from + 9.31 to + 13.91, and**(c)** Grand Average Scalp Topography of ΔP3b Across All Participants. *n* = 70).

Figure 1 depicts the relationship between bilingual language engagement (i.e., composite scores) and ΔP3b mean amplitude. For illustrative purposes of the ERP waveform, participants are binned into sub-groups based on +/- 2 standard deviations from the mean composite score. Group 1 (lowest bilingual experience; *n* = 15) with composite scores from − 7.05 to − 1.61; Group 2 (next lowest; *n* = 16) with scores from − 1.60 to + 3.85; Group 3 (moderately high; *n* = 26) with scores from + 3.86 to + 9.30; and Group 4 (highest; *n* = 13) with scores from + 9.31 to + 13.91. Scores in Group 1 reflect minimal non-English exposure with only receptive knowledge and limited use of a non-societal language across interlocutors and contexts. According to Anderson et al.^64^, individuals with composite factor scores between − 3.12 and + 1.22 can be classified as having ambiguous language backgrounds (i.e., cannot be classified as bilingual or monolingual) and are instead may reflect receptive bilinguals. See Figure S1 in the Supplementary Materials for scalp topographies of ΔP3b by bilingual language experience.

## Discussion

The goal of the present study was to investigate how variation in bilingual language experience modulates neural processing in young adults with diverse language backgrounds. We employed an active visual oddball task to elicit the P3b component, which is a well-established electrophysiological marker of attentional processing^33,34,38^. As expected, when collapsing across all participants regardless of language experience, robust task effects emerged: Rare Oddball trials elicited slower RTs and larger P3b amplitudes relative to Frequent Standard trials, confirming that the task successfully engaged the cognitive processes associated with attention allocation. We then examined whether differences in language experience accounted for variability in these effects while controlling for parental education serving as a proxy for childhood family SES. Although bilingual language engagement did not predict behavioral performance, it was significantly associated with the magnitude of the ΔP3b amplitude (Rare Oddball - Frequent Standard). Specifically, individuals with more bilingual language engagement demonstrated larger (more positive) ΔP3b responses, even after accounting for SES. Importantly, when testing variation in bilingual language engagement, we tested both linear and quadratic associations with respect to modulations in P3b amplitude. The results here supported a significant linear relationship, whereas the quadratic term was not significant, indicating no evidence for a U-shaped pattern. Therefore, our results suggest that bilingual experience may influence the neural efficiency of attentional resource allocation, thereby selectively modulating the neural aspects of task performance without impacting behavior.

To our knowledge, this is the first study to demonstrate an association between bilingual language experience and the P3b effect using an active visual oddball task. This distinction is important because prior research linking bilingualism to P3b modulation has relied primarily on other paradigms (e.g., Stroop, Flanker, Simon), which emphasize mechanisms of conflict monitoring, inhibitory control, and interference resolution^46–50,66,67^. Findings from those tasks have therefore been interpreted as evidence that bilingual experience influences executive control processes engaged during the detection and resolution of response conflict.

In contrast, our findings situate the P3b within a framework focused on attentional allocation^33,34^. The visual oddball paradigm does not require conflict resolution; rather, it indexes the neural allocation of attention toward infrequent, task-relevant events. By showing that greater bilingual language experience is associated with larger ΔP3b amplitudes in this context, our findings suggest that bilingual engagement modulates how attentional resources are recruited when salient information must be attended within a stream of ongoing input. Thus, instead of reflecting enhanced conflict monitoring or inhibitory control, the observed P3b modulation indicates that bilingual language experience shapes fundamental mechanisms of attention allocation during goal-directed processing.

Importantly, rather than reducing interpretability, our use of a visual oddball paradigm provides a valuable extension of the existing literature. By eliciting the P3b outside the context of response conflict, the present findings demonstrate that the influence of bilingual language experience is not limited to domains involving inhibitory control or conflict resolution but also encompasses broader mechanisms of attentional engagement. Employing different paradigms is essential for disentangling which aspects of cognition are most sensitive to the bilingual experience. Only through systematic comparison across tasks and across heterogeneous bilingual populations can we determine the range and specificity of bilingualism-related neural adaptations. In addition, our findings complement related work using passive oddball paradigms that target earlier or adjacent ERP components. For example, studies of the mismatch negativity (or MMN), an index of pre-attentive change detection that temporally precedes the P3a, have shown that bilingualism can influence early sensory-level processing^68–72^. Similarly, research examining the P3a component has highlighted the effect of bilingual experience on phonetic encoding (e.g.,^46,73,74^) and language-mediated visual object perception^46,75^. Together, these converging lines of evidence suggest that bilingual experience shapes neural processing across multiple stages of the information-processing stream, from early sensory discrimination to later attentional allocation indexed by the P3b.

Another strength of the present study is its characterization of bilingualism as a multidimensional continuum rather than a categorical distinction. This approach may help account for inconsistencies in P3b findings across studies varying in sample composition. Indeed, two relatively recent ERP/EEG studies illustrate how variation in bilingual engagement relates to neural indices of cognitive processing^62,63^. Jiao et al.^62^ found that Mandarin Chinese-English bilinguals with higher L2 proficiency exhibited larger N2 and P3b amplitudes during a spatial Stroop task, despite no behavioral differences, which they interpreted as reflecting more efficient neural resource allocation during conflict resolution. An alternative interpretation is that larger amplitudes may indicate compensatory effort^11^. Pereira Soares et al. took a broader approach, assessing multiple aspects of bilingual language experience in a mixed bilingual and multilingual sample. Although they found no ERP differences as a function of bilingual engagement on an N-back task, they observed robust individual-level associations in the oscillatory domain^63^. Pereira Soares et al. attributed their null ERP findings partly to characteristics of the N-back paradigm and proposed that variability in bilingualism-related effects may depend on sample composition, particularly whether analyses were restricted to bilinguals or included a broader monolingual-bilingual continuum. Consistent with this view, the present study, which included participants spanning the full bilingualism continuum, revealed that even subtle differences in cumulative bilingual engagement modulated neural indices of attentional engagement (ΔP3b amplitude) in the absence of behavioral effects, suggesting that both task demands and sample composition may account for discrepancies across studies.

The present findings also extend research that has largely focused on homogeneous bilingual samples or specific language contexts^11,17,44,45^. The sociolinguistic environment of bilinguals in the United States differs substantially from that of L2 learners in structured immersion settings (e.g., Mandarin-speaking students learning English in school). In the U.S., non-societal languages are often acquired informally at home or in community settings, while English dominates formal education and academic life^76,77^. This sociocultural configuration encourages frequent, context-dependent language switching, placing continuous demands on attentional systems that monitor, select, and adapt to rapid changes in language context. In contrast, bilinguals in formal immersion or classroom-only environments typically experience more compartmentalized language use, offering fewer opportunities for spontaneous switching. The present study’s results are consistent with the interpretation that a history of actively monitoring one’s language environment is associated with greater engagement of mechanisms underlying attentional allocation.

A central challenge for understanding the present findings is to specify why variation in bilingual language experience would modulate P3b amplitude during an active visual oddball task. This task requires participants to maintain task rules in working memory, categorize stimuli, and detect infrequent targets amid frequent standards, all while engaging attentional updating and managing resource allocation when rare events occur^36^. We propose that similar attentional mechanisms are continuously recruited in active bilingual communication, especially among individuals who frequently navigate dual-language environments. For instance, bilingual individuals’ ability to monitor the environment for potential language switches requires sustained attentional control and dynamic context updating, processes conceptually akin to detecting rare targets in an oddball stream. Over time, such experience may tune the neural systems supporting attentional engagement, leading to larger P3b amplitudes when salient, low-probability events occur. This interpretation aligns with Bialystok’s proposal^31,32^ that bilingual experience reshapes the attentional system, yielding measurable effects under conditions of heightened attentional demand. Accordingly, the relationship observed here between bilingual language experience and the P3b effect can be viewed as a process-specific bilingual adaptation, reflecting enhanced allocation of attentional resources to behaviorally relevant changes in the environment.

In interpreting the present results, it is important to note that although we observed the expected RT difference between Rare Oddball and Frequent Standard stimuli, bilingual language experience was not associated with variability in the magnitude of the ΔRT effect. This pattern mirrors findings from related work (e.g., ^62,63^), in which effects of bilingual experience were observed at the neural level but not in overt behavioral performance. One likely explanation concerns the characteristics of both the sample and the task. Young adults tend to perform near ceiling on simple discrimination tasks and are often at a developmental peak in processing efficiency^78,79^, particularly when tasks are low in complexity (e.g.,^35,61^). While neural systems that support attentional control continue to mature throughout this period^80^, our participants may have already reached asymptotic levels of cognitive performance through accumulated life experiences, including formal education and extensive academic task engagement^81^. As a result, subtle differences in attentional allocation may be detectable neurally but may not translate into measurable behavioral variation.

The interpretation of larger neural responses in the absence of behavioral differences remains an ongoing point of discussion in the field. Some researchers argue that larger P3b amplitudes without corresponding performance improvements may reflect compensatory effort or reduced processing efficiency^17^, whereas others contend that ERPs can reveal meaningful experience-dependent adaptations that behavioral metrics alone cannot capture^24,28^. Larger P3b responses may therefore reflect either the recruitment of additional neural resources to maintain performance or an enhanced sensitivity to salient, task-relevant events that do not necessarily impact accuracy or speed. In the present study, the neural data provide clear evidence of differences in attentional engagement as a function of bilingual language experience, highlighting the value of electrophysiological measures for detecting subtle cognitive adaptations that may not manifest behaviorally (cf.^82^). Future studies employing more demanding or behaviorally sensitive paradigms (e.g.,^83^) will be essential for determining whether these neural adaptations translate into functional performance advantages under conditions of greater attentional load.

Notably, in the present study, we observed a linear association between individuals’ composite scores and P3b amplitude, but not a non-linear (e.g., inverted U-shaped) relationship. This finding aligns with both the DRM and UBET frameworks’ predictions that neurocognitive adaptations correlate with bilingual language engagement. Specifically, the DRM proposes that bilingual brain adaptations occur in progressive stages over time: initial cortical expansion during early L2 learning, followed by consolidation with subcortical and white matter changes as experience increases, and finally peak efficiency with highly efficient allocation of cognitive resources^29^. These stages reflect increasingly efficient neural organization rather than a rise and fall in bilingual ability. Similarly, the UBET framework posits that different aspects of bilingual experience, including intensity and diversity of language use, language switching, relative proficiency, and duration of use, drive distinct but complementary neurocognitive adaptations^30^. According to the UBET framework, greater engagement with an additional language (reflected in higher composite factor scores in our sample) should progressively enhance cognitive processes rather than follow an inverted U-shaped function. Furthermore, because all participants are bound to the sociolinguistic context in the U.S., our sample has functional proficiency in English (required for study participation and task completion). Therefore, in our sample, composite scores reflect greater non-English language maintenance rather than reduced overall bilingual ability, making a U-shaped relationship theoretically implausible in this context.

We also observed that higher childhood parental education was associated with larger neural responses, independent of bilingual experience. Prior work indicates that SES can moderate relationships between bilingualism and cognitive processing^63^, and that early SES can exert lasting influences on neural function into adulthood^61^. However, the present sample was not large enough to fully assess potential interactions between bilingual experience and SES. Given the implications for understanding how linguistic and socioeconomic contexts jointly shape cognitive development, future research with larger, more demographically balanced samples will be necessary to determine whether childhood SES moderates the relationship between bilingualism and attentional processing.

Finally, our study relied on self-reported language proficiency, which may not fully capture the breadth of bilingual language competence. Although self-reports correlate with objective measures, future work should incorporate direct proficiency assessments, such as vocabulary tests (e.g., Multilingual Naming Test^84^, LexTALE^85,86^, as well as other subjective measures to index language entropy to elucidate how the balance and diversity of language use across contexts influences neural processing^84–87^. Furthermore, the present sample included speakers of multiple language types (e.g., Mandarin, Spanish, French) but without sufficient representation to facilitate meaningful group comparisons. Thus, future studies with larger, more balanced samples across tonal and non-tonal languages will be needed to elucidate whether structural language characteristics differentially relate to neural mechanisms of attentional allocation. Moreover, collecting richer sociodemographic information (e.g., immigration status, language exposure histories), as well as biological sex, should be done in future studies to more precisely characterize how contextual and biological factors, respectively, shape attentional mechanisms in bilingual individuals. Finally, although we observed a significant linear relationship between bilingual language engagement and attentional processing but not a non-linear effect, it is possible that non-linear effects of language experience on neural activity may emerge in samples with a broader distribution of language engagement or in different sociolinguistic contexts. Even so, the present findings underscore the importance of examining individual variation in bilingual language experience, aligning with recent calls to move beyond categorical group comparisons and toward continuous, context-sensitive approaches to understanding bilingualism and the brain^51,54,55,82,88,89^.

## Method

### Participants

Participants were recruited through flyers posted at the University of California, Merced campus and at community centers in Merced County. Inclusion criteria required participants to have normal or corrected-to-normal vision, no hearing problems, no history of brain injuries or neurological disorders, and no current use of medications that could alter brain activity. Participants provided written informed consent and were compensated $30 in cash for their participation. The University of California, Merced Institutional Review Board approved the study protocol (#UCM2022-42). As such, all methods of study were carried out in accordance with the guidelines and regulations of the Declaration of Helsinki. All study recruitment and task materials were administered exclusively in English. Participants (*n* = 83) were drawn from a larger study investigating associations between childhood family SES and adult neural 61processing^61^. The present study includes the subset of participants who completed the bilingual language questionnaire, as not all the participants in the larger study completed it. We excluded adults who completed the sociodemographic questionnaire incorrectly (i.e., reported the education completed by adults they currently live with, such as roommates, instead of their parents/legal guardians, *n* = 4), exhibited poor ERP data quality for the ERP task (*n* = 6), or achieved below chance (at or less than 50%) accuracy on any given condition in the visual oddball task (*n* = 3) from the analytical sample. Participants with and without usable ERP data did not differ based on age, participant education level, childhood parent education, or bilingualism factor scores (all *p* values ≥ 0.69) Among those excluded (*n* = 13), their degree of bilingualism (i.e., Composite Factor Score) ranged from − 5.26 to + 11.05 (M = 3.73, *SD* = 5.30), indicating that excluded participants did not cluster exclusively at the low or high ends of the bilingual language continuum. Furthermore, this distribution suggests that excluding these individuals did not systematically bias the sample or the interpretation of the findings. Table S3 provides a summary of descriptive statistics and an independent samples t-test comparing the sociodemographic characteristics of participants with usable and unusable ERP data (see Table S3 in Supplementary Materials).

The final analytical sample consisted of 70 adults who were 18 to 30 years old (M = 22.24, *SD* = 2.84). Of these participants, 57% identified as female, 41% as male, and 2% identified as non-binary. Biological sex data were not collected. The reported race/ethnicity of participants was as follows: 66% Hispanic or Latino/x, 10% Asian/Asian American, 11% selected more than one race/ethnicity, 11% White/European American, and 1% Middle Eastern. All participants reported completing high school. Among those with post-high school degrees, 11% held an Associate of Arts (AA) or Associate of Science (AS) (a two-year postsecondary degree typically awarded by U.S. community colleges), 14% held a bachelor’s degree, and 3% held a master’s degree. Four participants reported knowledge of only English, while 66 reported knowledge of English along with an additional language: Spanish (52), Chinese dialects (not specified by the participant) (3), Mandarin (3), Japanese (2), French (2), Italian (2), Indonesian (1), and German (1).

### Measures

The language social background questionnaire (LSBQ) was adopted to collect participants’ language history^64^. To prevent confusion and misuse of the “non-applicable” option, a trained researcher administered the questionnaire before the active visual oddball task. The LSBQ consists of three sections: (1) personal background, (2) languages spoken and language use, and (3) language use in context. Due to the inclusion of a separate demographic questionnaire in this study, we omitted items from the personal background section (e.g., age, occupation, and education).

In the languages spoken and language use section, participants first identified the languages they could speak and understand. Participants self-reported their proficiency levels for speaking, writing, reading, and listening in English and any additional non-English language (e.g., Spanish) on a scale from 0 (no proficiency) to 10 (high proficiency). Adults also reported the frequency of language use behaviors in English and the additional language using a 5-point Likert scale (0 = only English, 2 = half English/half non-English language, 4 = only the non-English language). This section gathered information about language use across various contexts (i.e., home, school, shopping centers), developmental stages (i.e., infancy, preschool age, and high school age), and activities (e.g., reading, watching TV, and listening to music), as well as with different speakers (e.g., partner, friends, neighbors). The final section assessed the frequency of language-switching behaviors when interacting with parents, friends, and on social media platforms. Three items indexed language switching. For example, participants rated how often they switched between English and a non-English language within the same conversation when talking with friends. Responses were scored on a 5-point Likert scale ranging from 0 (never) to 4 (always).

Based on the factor analysis conducted by Anderson et al.^64^ bilingualism was characterized using three sub-factors: (1) Social Use Score, (2) Home Use/Non-English Proficiency Score, and (3) English Proficiency Score. Each participant’s sub-scores were standardized and multiplied by the corresponding factor weights reported by Anderson et al.^64^. This analytic approach and factor weights are identical to those in the factor analysis conducted by Anderson et al.^64^. These weighted standardized scores were then summed to derive a Composite Factor Score, indexing overall bilingual language experience. Larger (more positive) composite scores indicate greater bilingual language experience. Social Use scores reflect the use of a non-societal language (i.e., in our dataset any language other than English) in social contexts (e.g., school, work, with friends, watching movies/TV). Larger (more positive) Social Use scores indicate greater use of a non-societal language in social contexts. Home Use/Non-English Proficiency (hereafter Home Use/Proficiency) scores index the use of a non-societal language in home contexts and proficiency in that language (e.g., Spanish, Mandarin, French). Larger (more positive) Home Use/Proficiency scores indicate higher proficiency and greater use of that language in home contexts (e.g., language use with grandparents, parents, siblings). English Proficiency scores reflect subjective language proficiency levels in English, with more negative scores indicating greater proficiency.

Importantly, in English-dominant environments like our university, English proficiency tends to show relatively low variability among individuals compared to use of a non-societal language. Because the composite score is computed as a weighted combination of latent factors based on the variance explained by each factor, in our sample, it tends to be more sensitive to differences in the use of a non-English language and proficiency in that language (i.e., Mandarin, Spanish) than in the use of English. Thus, higher composite scores reflect overall bilingual language engagement, particularly increased proficiency and use of a non-societal language, rather than lower English proficiency. Indeed, if two individuals have equal non-societal language skills but differ in English proficiency, the individual with higher English proficiency would receive a higher composite score. Distributions of all factor scores (Composite, Social Use, Home Use/Proficiency, and English Proficiency) are shown in Figure S2 in the Supplementary Materials. Zero-order correlations revealed that the composite factor score and sub-scale language use factor scores (i.e., Social Use, Home Use/Proficiency scores) were highly correlated with one another (*r* values ≥ 0.70, all *p* values ≤ 0.001). However, the English proficiency factor was not significantly associated with the composite or language use factor scores (*p* values ≥ 0.17). Accordingly, only the overall degree of bilingualism factor score (i.e., Composite Score) was used in the analyses reported here. Exploratory analyses also examined the effects of either the Social Use or Home Use/proficiency sub-scale language use factor scores on ΔRT and ΔP3b amplitude and revealed the same pattern of results as the Composite Score analyses (see Table S4 in Supplemental Materials). Hence, we did not include these additional analyses in the present report.

Participants reported their age, gender, race/ethnicity, and childhood parental education, which served as a proxy for objective childhood family SES. Participants completed the SES questionnaires after the ERP task was completed. To capture childhood family SES, participants were prompted to recall their 10-year-old selves and indicate the highest level of education attained by at least one parent or legal guardian (e.g., mother, father, grandparent; henceforth referred to as ‘parent’). Parental education is commonly used as a proxy for childhood family SES in adult samples, as it reflects relatively stable aspects of socioeconomic background, is reliably reported retrospectively, and is associated with cognitive outcomes in later life^90^. While retrospective reports may introduce some measurement error, parental education is considered less vulnerable to recall bias than income-based measures and remains a widely accepted indicator of early-life SES^91–95^.

Educational attainment categories followed those used in the Survey of Income and Program Participation^96^. For analyses, we converted these categories into years of education completed, ranging from 0 (less than 1st grade) to 20 years (graduate or professional degree). Refer to the Appendix (in the Supplementary Materials) for the SES questionnaire and coding scheme.

Maternal education was strongly correlated with the highest parental education completed (*r* = .87). To preserve family SES data for participants from diverse family structures (e.g., where an uncle serves as a primary legal guardian), we used the highest level of parental education as a proxy for family education instead of solely relying on maternal education. We found the distribution of reported parental education levels to be as follows: 30% had less than a high school diploma, 23% had a high school diploma, 14% had some college but no degree completed, 8% had an associate’s degree (a two-year postsecondary degree typically awarded by U.S. community colleges), 13% held a bachelor’s degree, 17% had a master’s degree, and 4% had a professional degree or doctorate. On average, parents completed twelve years of education (M = 12.11, *SD*: 4.30), indicating that at the time that the participants were 10 years old, their parents had not pursued additional education beyond high school (see Figure S3 in the Supplementary Materials for the distribution of childhood parent education).

### Active visual oddball task

The active visual oddball task used in this study was obtained from ERP CORE (Compendium of Open Resources and Experiments), which provides paradigms optimized for studying various ERP components^35^. The task was presented using Presentation software (Neurobehavioral Systems, Inc., Berkeley, CA). Visual stimuli were displayed on a medium gray background (x = 0.35, y = 0.36, 25.9 cd/m^2^) on a Dell LCD monitor with a resolution of 1280 × 1024, a refresh rate of 60 Hz, and a viewing distance of 95 cm. The visual paradigm included a white fixation point (0.15º visual angle) at the center of the screen. Participants were instructed to maintain fixation on this point throughout the task and to withhold blinking or moving their eye gaze until after a button response. Verbal instructions were given to the participant before the start of the task, and written instructions were presented on the screen during the task. Participants used a game controller to respond and had the option to take breaks after each block, allowing them to control the duration and frequency of their breaks.

Except for the method used to gather behavioral responses (i.e., a keyboard for ERP CORE and a game controller in the present study), this task matched the ERP CORE version. On each trial, a single capitalized letter from a set of five letters (A, B, C, D, or E) in the Geneva font was displayed on the screen for 200 ms, covering a 2.5º x 2.5º visual angle. Sequential stimuli were shown superimposed on the fixation point and separated by a jittered interstimulus interval randomly varying between 1200 and 1400 ms. Participants completed 200 trials divided into five blocks.

In each block, four letters served as non-target stimuli (Frequent Standard, 80% probability), and one letter served as the target stimulus (Rare Oddball, 20% probability). Therefore, the target letters themselves constituted the Rare Oddball events, and there were no additional frequency manipulations. Across the five blocks, each letter (A, B, C, D, E) served as the Rare Oddball target in one block, with block-wise target assignment counterbalanced within each participant. Using the game controller, participants pressed the “up” or “down” buttons to indicate whether the presented stimulus was a target (Rare Oddball) or a non-target (Frequent Standard) letter. Participants were not explicitly informed of the difference in occurrence probability between the target (Rare Oddball) and non-target (Frequent Standard) letters.

In the visual oddball task, trials with RTs that were either faster than 200 ms (anticipatory responses) or longer than 1000 ms (signaling lapses in attention) were excluded from behavioral and ERP data analyses, consistent with ERP CORE^35^. Although we calculated each participant’s discriminability index *d*′ = Z(Correct/Hit) – Z(Incorrect/False Alarm)^97^, accuracy was at ceiling. As a result, we focused our behavioral analyses on RTs. Only trials with correct responses were included in RT analyses. Specifically, we computed an ΔRT effect (i.e., Rare Oddball median RT - Frequent Standard median RT) to mirror the P3b effect.

### Electroencephalogram (EEG) recording

**Fig. 2:**
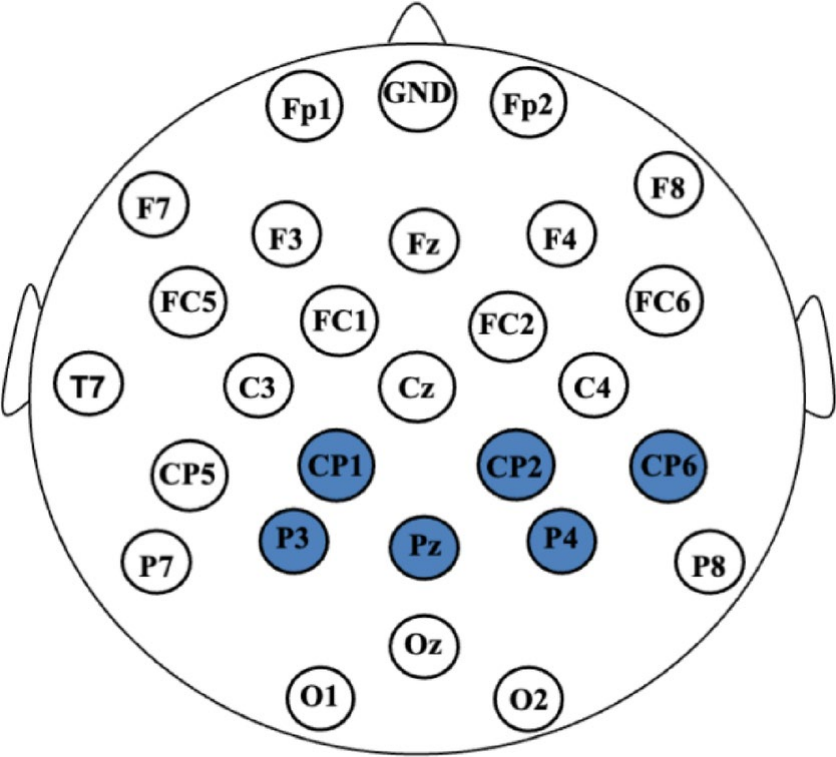
Configuration of the Scalp Electrodes. The posterior electrodes (Pz, P3, P4, CP1, CP2, CP6), highlighted in dark blue, served as the region of interest for the P3b analyses. The ground electrode was positioned at Fpz (GND), and Cz served as the online reference.

The EEG data were recorded using the Brain Products actiCHamp Plus system and BrainVision Recorder Version 1.25.0101. The EEG data were referenced to Cz and sampled at 500 Hz during recording. The actiCAP slim active electrode system, mounted on elastic snap caps, was used to collect the data (actiCHamp Plus, Brain Products GmbH, Gilching, Germany). Fpz was the location of the ground electrode. Five electrodes from the 32-channel electrode bundle were repurposed for offline re-referencing and electrooculogram (EOG) recording. Two electrodes (TP9 and TP10) were placed over the mastoid bones behind the left and right ears. To record the EOG, three additional electrodes (FT9, FT10, and T8) were repurposed. The horizontal EOG (HEOG) electrodes were positioned lateral to each eye’s external canthus, and the vertical EOG (VEOG) electrode was positioned beneath the right eye. The remaining 27 electrodes were mounted in line with the international 10/20 system and utilized as scalp electrodes (see Fig. 2 for the electrode configuration). StimTrak (Brain Products, Gmbh, Gilching, Germany) was used to measure the stimulus presentation delay in the monitor. The visual stimulus was delayed by approximately 20 ms.

### EEG/ERP processing and analyses

Using customized EEGLAB^98^ and ERPLAB^99^ scripts, as well as scripts modified from ERP CORE^35^, and Independent Components (IC) Label^100^, EEG signal processing and averaging were carried out in MATLAB. The data processing and analysis pipeline employed in the current study is consistent with that used in a previous study with young adults^61^. All scripts are available on Open Science Framework: https://osf.io/3aczu/.

EEG data were re-referenced to the arithmetic average of the left and right mastoids. Bipolar eye channels were constructed to detect ocular artifacts: the horizontal bipolar eye channel was calculated by subtracting the left HEOG from the right HEOG and the vertical bipolar eye channel was computed by subtracting Fp2 from the right VEOG. The finite impulse response filter in EEGLAB was used to band-pass filter the EEG data between 0.1 and 40 Hz (with a -6 dB cutoff frequency).

To remove EEG artifacts, we employed a combination of independent component analysis (ICA) and trial rejection based on residual artifacts. To prepare the data for ICA, we removed recording intervals that did not contain event codes, which were defined as periods without event codes lasting 6000 ms or longer, with a 3000 ms buffer between and after any event codes. We applied the ERPLAB moving window peak-to-peak threshold algorithm over a 500 ms window duration, moving in 50 ms increments, with a +/- 300 µV threshold to all the scalp channels to identify bad channels that would lead to excessive data loss. Apart from Fp1 and Fp2, any scalp channel that led to substantial data loss was excluded from pre-ICA artifact rejection. Substantial data loss was defined as exceeding 10% overall data loss and ≥ 3.29 *SD* data loss compared to other scalp channels for each participant^101^. Subsequently, the ERPLAB moving window peak-to-peak threshold algorithm was applied to reject data segments that contained extreme artifacts from the continuous data. This algorithm operated within a 500 ms window, moving in 50 ms increments, and applied a +/- 300 µV threshold to all scalp channels, except for Fp1 and Fp2.

ICA was implemented on all channels, excluding the bipolar eye channels. The calculated ICA weights were then applied to the preprocessed data files (i.e., prior to pre-ICA cleaning). Artifact correction involved removing components designated as “eye” components if ICLabel classified them as “eye” with a confidence level of at least 80% and as “brain” with a confidence level of less than 5%. These criteria were established based on a systematic comparison of various confidence thresholds for young adults.

Prior to epoching, the stimulus event codes were adjusted in time by 20 ms to compensate for the visual stimulus presentation delays mentioned earlier. Following ERP CORE parameters, ICA-corrected data were epoched from − 200 to + 800 ms relative to stimulus onset, with the − 200 to 0 ms pre-stimulus interval used for baseline correction. To eliminate any residual eye artifacts, ICA-corrected bipolar HEOG and VEOG channels were created. To prevent unnecessary data loss from a faulty channel, a simple voltage threshold algorithm with a +/- 200 µV threshold was first applied to all the scalp channels of analytical interest. Channels were excluded if they resulted in over 10% trial loss and a data loss rate of at least 3.29 SD^101^ above the other channels. None of the channels of interest met these exclusion criteria.

To identify trials with residual artifacts in the scalp channels of interest (Fz, F3, F4, FC1, FC2, FC5, FC6, C3, C4, CP1, CP2, CP5, CP6, Pz, P3, P4, Oz, O1, O2), we used a simple voltage threshold algorithm with a +/- 200 µV threshold, along with a moving peak-to-peak window algorithm that moved in 100 ms intervals and applied a 125 µV threshold to each epoch. To remove residual blinks and saccades, we applied a moving peak-to-peak window algorithm on the ICA-corrected VEOG, using a 200 ms window that moved in 50 ms increments with a 150 µV threshold, and a step-like algorithm was applied to the ICA-corrected HEOG, using a 100 ms window that moved in 10 ms increments with a 64 µV threshold. A peak-to-peak window algorithm (between − 25 to + 225 ms relative to stimulus onset, moving at 10 ms increments, with a 150 µV threshold) was applied to the uncorrected VEOG to reject trials where blinks occurred too close to the presentation of visual stimuli. Additionally, to remove trials with saccades occurring too close to the visual stimuli presentation, we used a step-like algorithm on the uncorrected HEOG (between − 50 to + 250 ms relative to stimulus onset, moving at 10 ms increments with a 32 µV threshold). In line with ERP CORE, trials with incorrect behavioral responses and RTs faster than 200 ms or slower than 1000 ms were excluded from the analyses. These behavioral criteria resulted in an average exclusion rate of 1.43% for Frequent trials and 13.2% for Rare trials across participants. Subsequent EEG artifact rejection resulted in the removal of an additional 6.19% of remaining Frequent trials and 5.43% of remaining Rare trials. No channels required interpolation. ICA resulted in the removal of an average of 2.16 components per participant (SD = 0.53, range = 0–3).

### EEG/ERP data quality

Following artifact rejection, we visually examined individual ERP plots for data quality. Additionally, to quantify ERP data quality and measurement precision, we computed analytic standardized measurement error (aSME) and bootstrapped standardized measurement error (bSME), which provide estimates of the reliability of ERP amplitude measurements^102^. The SME reflects the expected variability in ERP amplitude estimates due to noise and trial counts, with smaller SME values indicating greater measurement precision and higher data quality. The aSME is calculated analytically based on within-participant trial variability and number of trials, whereas the bSME is estimated using bootstrapping procedures that resample trials to empirically quantify measurement precision. Both metrics provide complementary estimates of the standard error of ERP amplitude measurements and are considered good practice approaches for evaluating ERP data quality^102^ across studies.

In general, lower SME values indicate more precise and reliable ERP estimates, and SME values should be interpreted relative to the magnitude of the ERP effect of interest, with smaller SME relative to the observed ERP amplitude reflecting a higher signal-to-noise ratio and more reliable measurements. We note that cutoff values for ERP data quality measures, including aSME and bSME, are not described, since these measures depend on dataset-specific factors (e.g., overall variance and sample size)^102^. Nonetheless, these ERP data quality metrics provide useful information about data precision in ERP studies^102^. The descriptive statistics for the quantity of artifact-free ERP trials, accuracy rates, ERP mean amplitudes for the observed waveforms, aSME values for the Frequent Standard and Rare Oddball conditions, and bSME values for the difference wave (i.e., ΔP3b) reported in Table S5 (see Supplementary Materials).

Two additional participants were excluded because their data failed to show discernible visual evoked potentials. Importantly, these two participants were not among those previously excluded due to missing demographics, subpar behavioral performance, or noisy EEG data. As a result, the final analytical sample included 70 participants.

### Cluster-based permutation test

We conducted a cluster-based permutation test using the Field Trip package^103,104^ to provide empirical justification for our choice of electrodes included in the main statistical analyses. To do this, we contrasted the ERPs from the Rare Oddball and Frequent Standard trials (i.e., only the trials with a correct response) via within-participant contrasts across the entire sample, regardless of language experience. ERP data from the 26 scalp electrodes of interest (excluding EOG and mastoid electrodes) were inputted into the cluster-based permutation test. First, dependent-samples t-tests were conducted at each time-channel data point for both the actual data and for the permuted data (1,000 permutations). Next, clusters of adjacent time-electrode data points which had significantly different ERP amplitudes between the two conditions (based on the t-tests) were formed. Initially, cluster formation included any time-channel points with a univariate t-test *p* value of < 0.05; however, due to the strong and widespread resulting P3b effect, the *p* value threshold for cluster formation was reduced to < 0.001. The same cluster formation procedure was applied to each permutation (i.e., random shuffling of the condition labels) of the data to create a null distribution, and Field Trip’s ‘maxsum’ algorithm was used to compute Monte Carlo cluster-level *p* values (see Figure S4 in Supplemental Materials for a summary of the results).

The cluster-based permutation tests revealed that the Rare Oddball trials had a significantly greater P3b amplitude than the Frequent Standard trials as expected (cluster-level *p* value = 0.0020). This effect was observed across all 26 scalp electrodes, with the strongest P3b effect observed over parietal and posterior scalp sites. We then sorted the electrodes from highest to lowest effect size (based on the mean t-statistic across all the time points that encompassed the P3b effect in that electrode), and the ten electrodes with the strongest effect were: P3, P4, Pz, CP2, CP6, CP1, O2, C4, Oz, P7.

In light of the permutation test results, we conducted several versions of the multiple regression model to predict the ΔP3b amplitude from participants’ bilingual composite scores and childhood parental education. Specifically, these models only differed in the electrodes used to extract mean ΔP3b amplitude, with one model using data from just the Pz, P3, and P4 electrodes (as is commonly done in the literature since the P3b effect is maximal over these electrodes^61^, a model using the top six electrodes with the strongest P3b effect from the general permutation test (P3, P4, Pz, CP2, CP6, and CP1), a model using the top ten electrodes with the strongest P3b effect from the general permutation test (P3, P4, Pz, CP2, CP6, CP1,O2, C4, Oz, and P7), and another model using all 26 scalp electrodes of interest (Fp1, Fp2, Fz, F7, F8, F3, F4, FC5, FC1, FC2, FC6, T7, C3, C4, CP1, CP2, CP5, CP6, P7, P3, Pz, P4, P8, O1, Oz, and O2). Across all models, the pattern of results was the same: both bilingual composite score and childhood parental education were significant predictors of ΔP3b amplitude, suggesting that the results are robust regardless of which electrodes are used to compute mean ΔP3b amplitude (see Table S6 in the Supplemental Materials for a summary of the results). This was also true for models that included the quadratic composite score term (i.e., composite score^2^, where bilingual composite score and childhood parental education were significant predictors of ΔP3b amplitude, but the quadratic term was not. Consequently, all subsequent analyses were conducted using the centroparietal cluster (P3, P4, Pz, CP2, CP6, and CP1), corresponding to the scalp distribution where the P3b is typically maximal^36,37^(see Figure S5 in the Supplemental Materials for the grand average plot over the centroparietal cluster across all participants).

### ERP time-window mean amplitude measures

ERP time-window mean amplitudes for P3b were measured based on the time window suggested in ERP CORE, which spans from 300 to 600 ms post stimulus onset^35^. We computed difference waves (Rare Oddball-Frequent Standard, or Δ) to isolate the experimental effect since difference waves remove common neural processing that is shared across conditions^105^. We used bSME scores as an objective data quality metric for the P3b difference wave^102^. Larger ΔP3b amplitude scores suggest larger neural responses to the Rare Oddball targets (more positive in amplitude) than the Frequent Standard non-targets. Only correct trials were included for behavioral and ERP analyses.

### Data analysis

Preliminary analyses were conducted for each variable of interest to check for univariate outliers. Any score above or below 3.29 *SD* beyond the mean was considered an outlier^101^. There were no outliers for childhood parent education levels, overall bilingual language experience scores (i.e., composite scores), *d*′, RTs (Rare Oddball and Frequent Standards), or the mean amplitude and standardized measurement error scores for P3b amplitudes.

Two multiple regression analyses were performed to examine how variation in bilingual language engagement shapes attention allocation (i.e., ΔRT Effect and ΔP3b amplitude). Both models included childhood parent education as a covariate to account for individuals’ socioeconomic backgrounds. In addition, a quadratic bilingual composite score term was included to test for potential (U-shaped) links between language experience and attention. All predictors (bilingual composite score, quadratic composite score, and childhood parent education) were mean-centered and scaled before analysis. Only correct trials were included in the behavioral and ERP analyses. The ΔP3b amplitude was computed as the difference between Rare Oddball and Frequent Standard trials, averaged across six centroparietal electrodes showing the strongest general task-related effects selected based on the cluster-based permutation test.

All scripts used in the analyses are accessible on Open Science Framework: https://osf.io/3aczu/.

1. The first regression examined the association between variation in bilingual language engagement and overall behavioral performance (ΔRT Effect ~ Childhood Parent Education Levels + Bilingualism Composite Score + Bilingualism Composite Score^2^.
2. The second regression assessed the relationship between variation in bilingual language engagement and ΔP3b amplitude (ΔP3b ~ Childhood Parent Education Levels + Bilingualism Composite Score + Bilingualism Composite Score^2^.

To control the familywise error rate, a Bonferroni correction was applied, resulting in a corrected alpha level of 0.025 (0.05/2). All statistical analyses were conducted using the lm() function in R (Version 4.3.1; R Core Team, 2023) and RStudio (Version 6.2.5621; RStudio Team, 2023). The datasheet used in the analyses and data dictionary are accessible to view on Open Science Framework: https://osf.io/3aczu/.

## Data Availability

The dataset supporting the conclusions of this article is available in the Open Science Framework repository,https://osf.io/3aczu/overview.
